# Screening of the HBx transactivation domain interacting proteins and the function of interactor Pin1 in HBV replication

**DOI:** 10.1038/s41598-021-93584-z

**Published:** 2021-07-08

**Authors:** Qiaoxia Zhou, Libo Yan, Baofu Xu, Xue’er Wang, Xuehong Sun, Ning Han, Hong Tang, Feijun Huang

**Affiliations:** 1 Department of Forensic Pathology, West China School of Preclinical and Forensic Medicine, Sichuan University, No. 17 Third Renmin Road North, Chengdu, 610041 People’s Republic of China; 2 Center of Infectious Diseases, West China Hospital of Sichuan University, No.37 Guo Xue Xiang, Chengdu, 610041 People’s Republic of China; 3 Division of Infectious Diseases, State Key Laboratory of Biotherapy, Sichuan University, Chengdu, 610041 People’s Republic of China; 4 Xinxiang Key Laboratory of Forensic Science Evidence, School of Forensic Medicine, Xinxiang Medical University, Xinxiang, 453003 People’s Republic of China

**Keywords:** Proteomics, Protein-protein interaction networks, Hepatitis B

## Abstract

Hepatitis B virus (HBV) X protein (HBx) has been determined to play a crucial role in the replication and transcription of HBV, and its biological functions mainly depend on the interaction with other host proteins. This study aims at screening the proteins that bind to the key functional domain of HBx by integrated proteomics. Proteins that specifically bind to the transactivation domain of HBx were selected by comparing interactors of full-length HBx and HBx-D5 truncation determined by glutathione-S-transferase (GST) pull-down assay combined with mass spectrometry (MS). The function of HBx interactor Pin1 in HBV replication was further investigated by in vitro experiments. In this study, a total of 189 proteins were identified from HepG2 cells that specifically bind to the transactivation domain of HBx by GST pull-down and subsequent MS. After gene ontology (GO) analysis, Pin1 was selected as the protein with the highest score in the largest cluster functioning in protein binding, and also classified into the cluster of proteins with the function of structural molecule activity, which is of great potential to be involved in HBV life cycle. The interaction between Pin1 and HBx has been further confirmed by Ni^2+^-NTA pulldown assay, co-immunoprecipitation, and immunofluorescence microscopy. HBsAg and HBeAg levels significantly decreased in Pin1 expression inhibited HepG2.2.15 cells. Besides, the inhibition of Pin1 expression in HepG2 cells impeded the restored replication of HBx-deficient HBV repaired by ectopic HBx expression. In conclusion, our study identified Pin1 as an interactor binds to the transactivation domain of HBx, and suggested the potential association between Pin1 and the function of HBx in HBV replication.

## Introduction

Hepatitis B virus (HBV) infection is one of the most important risk factors of hepatocellular carcinoma (HCC)^1–3^. As a member of the Hepadnaviridae family, HBV is closely related with pronounced host specificity and involves in acute and chronic liver infections^4^. The viral genome is a 3.2 kb, partially double-stranded, circular DNA, which consist of 4 open reading frames (ORFs) named pre-C/C, pre-S/S, P, and X, respectively^5^. Among these ORFs, the X ORF is responsible for encoding HBx protein^6^, which is considered to be a multifunctional regulator and one of the most important factors that involved in the pathogenesis and carcinogenesis of HBV^7^.

The importance of HBx in the transcription and replication of HBV has been determined in previous studies. HBx deficient HBV genome were unable to launch productive infections in woodchuck livers^8^. In vivo and in vitro studies have proved that exogenous transfected HBx has the ability to revert the transcription and replication of X-deficient HBV replicon to wild-type levels^9–11^. Besides, Tang et al. figured out the two-thirds of HBx C-terminal (containing amino acids 51 to 154) as the functional region of HBx, which contains the transactivation domain, while the N-terminal (containing amino acids 1 to 50) is not required in HBx augmenting HBV transcription and replication^12,13^.

As a transcriptional co-activator, HBx has been reported not to bind DNA directly. The transcriptional functions mainly rely on the interaction between HBx and other factors in host cells. Thus, screening the host proteins that may interact with HBx can help to understand the mechanism of HBx enhancing HBV transcription and replication, and are of great significance for finding targets to eliminate HBV replication and pathogenicity. Efforts have been made, methods such as yeast two-hybrid system^14,15^, TAP followed by MS (TAP/MS)^14,16^ analysis, GST pull-down^17–19^, and co-immunoprecipitation (Co-IP)^20,21^ has been widely applied to researches regarding the identification of HBx interactors.

Previous study identified several groups of proteins that interact with full-length HBx by GST pull-down^17^, and other studies mainly verified the proteins that may bind to HBx one by one. However, knowledges regarding the proteins that bind to the key functional domain of HBx are still insufficient. This study aims at screening the potential host proteins that may specifically interact with the key function domain of HBx systematically. According to the results of glutathione-S-transferase (GST) pull-down assay and subsequent mass spectrometry (MS), we compared the two groups proteins bind to full-length HBx and HBx-D5 truncation which doesn’t contain the functional region, respectively. Finally, 189 proteins that specifically bind to the transactivation domain of HBx were identified, among which we selected Pin1 as an important host factor and further investigated the role of Pin1 in HBV replication.

## Results

### Profiling of proteins interacting with HBx

**Figure 1:**
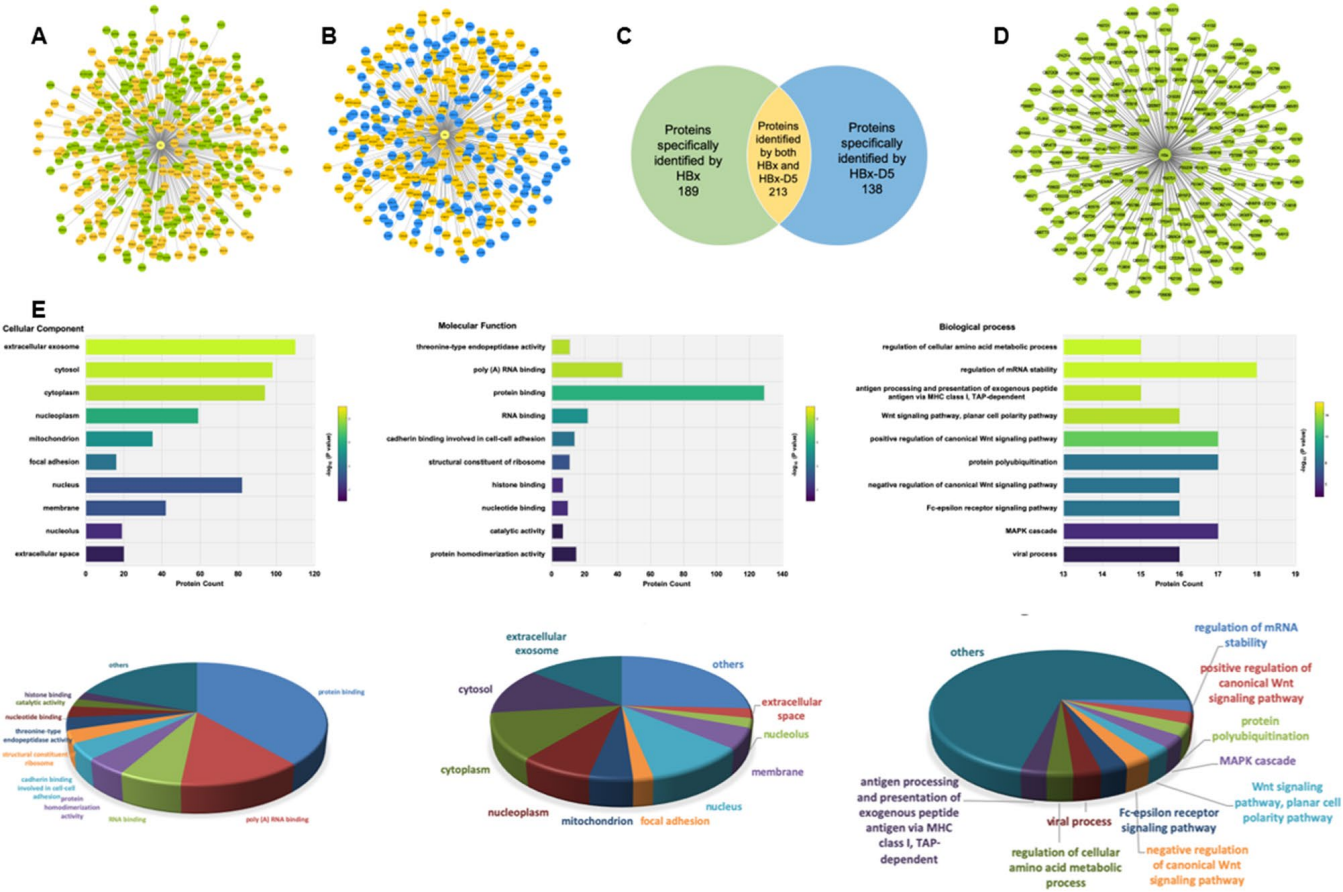
HBx interactome network and bioinformatic anlaysis. By comparing HBx interacted proteins (**A**) and HBx-D5 interacted proteins (**B**), proteins that specifically binds to HBx but not HBx-D5 were selected (**C** and **D**). Gene ontology analysis was conducted using the Database for Annotation, Visualization and Integrated Discovery (DAVID) (**E**).

GST pull-down screening was performed to analyze the potential proteins interacts with full-length HBx or HBx-D5 truncated HBx. The GST protein, GST-HBx, and GST-HBx-D5 expressed in *E. coli* were purified by GST agarose affinity chromatography (Supplementary Figure 1). Purified proteins were incubated with HepG2 cell lysate and glutathione-agarose beads. Samples of elution from three parallel experiments for each group were combined together and separated with SDS-PAGE gel following silver staining. Visible differences were detected between the GST lane, GST-HBx lane, and GST-HBx-D5 lane (Supplementary Figure 2), which were further confirmed by mass spectrometry analysis. Finally, a total of 402 proteins were identified by full-length HBx (Fig. 1A) and 351 proteins were detected to potentially interact with HBx-D5 truncation (Fig. 1B) after compared with GST protein to exclude non-specific bindings.

**Figure 2:**
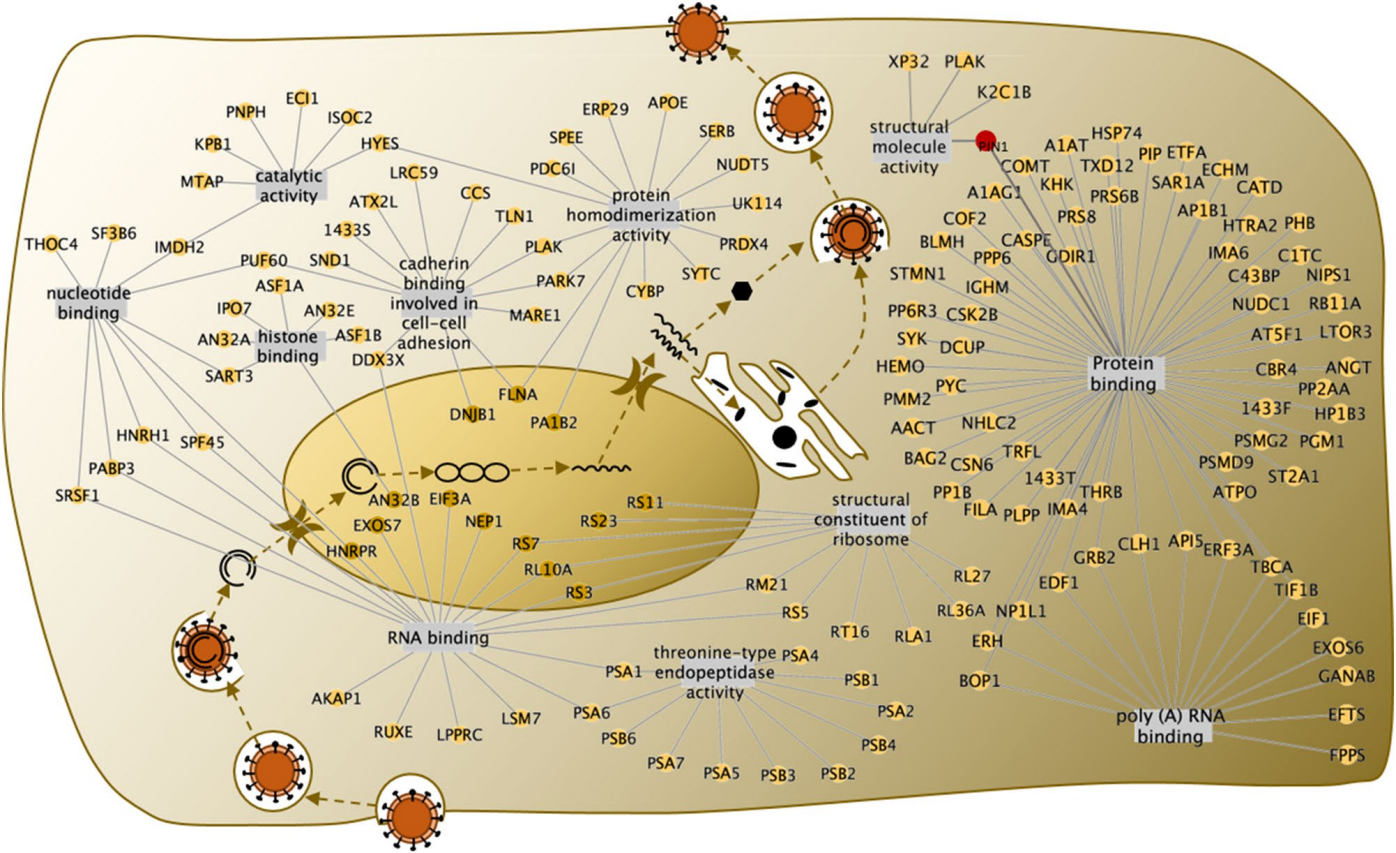
The identified HBx-interacting proteins that were possible to be involved in the HBV virus life cycle.

Comparing the two groups of proteins, we filtered out 189 proteins that interact with full-length HBx but not belong to the HBx-D5 identified group (Fig. 1C,D), which refer to those bind to the transactivation domain of HBx and have great potential to affect the transactivation and replication of HBV. To further investigate the functions of the HBx interacted proteins, an enrichment analysis was carried out using DAVID online software (https://david.ncifcrf.gov/home.jsp). A total of 75 biological process terms were identified from Gene Otology (GO) analysis (Supplementary Table 1), such as regulation of mRNA stability, regulation of cellular amino acid metabolic process, Wnt signaling, NIK/NF-kappaB signaling, and so on. 28 significantly enriched molecular function terms were analyzed (Supplementary Table 2), such as protein binding, RNA binding, poly(A) RNA binding, structural molecule activity, and so on (Fig. 1E). Thus, except participating in the cell signaling pathways, this group of proteins specifically identified by HBx transactivation domain have high potential to participate in the life cycle of HBV, including transcription of virus, protein translation, assembly of the virus capsule, and so on (Fig. 2). Among these interactors, Pin1 attracted our attention with the highest score in the largest cluster functioning in protein binding. As a member of peptidyl-prolyl *cis/trans* isomerase family, Pin1 was also reported to participate in various cell signal pathways and transcriptional regulation. Besides, Pin1 was classified into the cluster of proteins with the function of structural molecule activity as well. Considering that HBx is easily to degrade with a short half-life, Pin1 protein, which could contribute to the structural integrity of proteins, is of great potential to affect the life cycle of HBV virus.

### Verification of the interaction between HBx and Pin1

**Figure 3:**
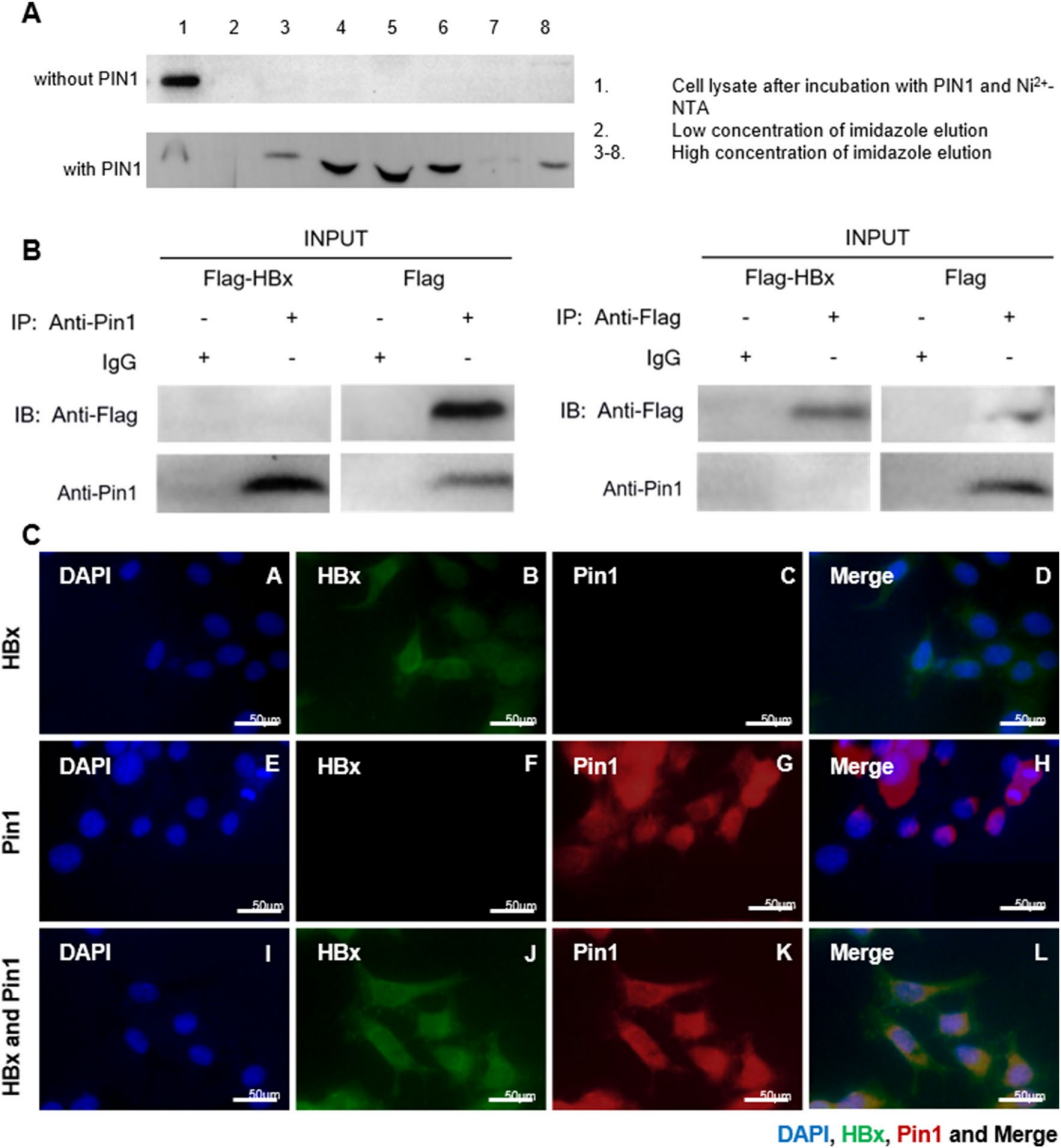
The interaction between HBx and Pin1. Cell lysate of BL21 transfected with Flag-tagged HBx expression plasmids was incubated with Ni2 + -NTA beads adding or not adding purified His_6_-tagged Pin1. HBx was detected in both the whole cell lysate isolated from Ni^2+^-NTA beads and imidazole eluent (**A**). Co-immunoprecipitation assay showed that with the transfection of Flag-tagged HBx, endogenous Pin1 protein pulled down by HBx was detected in eluent after incubated with anti-Flag beads, while HBx was detected in eluent after incubated with anti-Pin1 beads (**B**). Immunofluorescence microscopy shows the common position of Pin1 and HBx in both cytoplasm and nucleus (**C**).

The interaction between Pin1 and HBx was first confirmed using Ni^2+^-NTA pull-down assay. His_6_-tagged Pin1 and Flag-tagged HBx expression plasmids were transfected into BL21 cells respectively and the cell lysates were incubated with Ni^2+^-NTA to pull-down the His_6_-tagged Pin1 protein. HBx was detected in whole cell extract as well as the eluents of the bead. As shown in Fig. 3A, HBx expression was detected in both the whole cell lysate isolated from Ni^2+^-NTA beads and high concentration of imidazole eluent, but not detected in low concentration of imidazole fluent which was for elution of unbound proteins and non-specific binding. No HBx was detected in eluent without Pin1 incubation, suggesting that HBx could interact directly with purified Pin1 in vitro. Using co-immunoprecipitation assay, the interaction between HBx and Pin1 was further confirmed in a cellular context of HepG2 cells. With the transfection of Flag-tagged HBx, endogenous Pin1 protein pulled down by HBx was detected in eluent after incubated with anti-Flag beads. In contrast, HBx was detected in eluent after incubated with anti-Pin1 beads (Fig. 3B). These results indicated the direction interaction between HBx and Pin1 in HepG2 cells. To further address the physiologically relevant interaction, immunofluorescence microscopy was conducted. The results showed the common position of Pin1 and HBx in both cytoplasm and nucleus, which further confirmed the possibility of the interaction of Pin1 and HBx in HepG2 cells (Fig. 3C).

### Role of Pin1 in HBV replication

**Figure 4:**
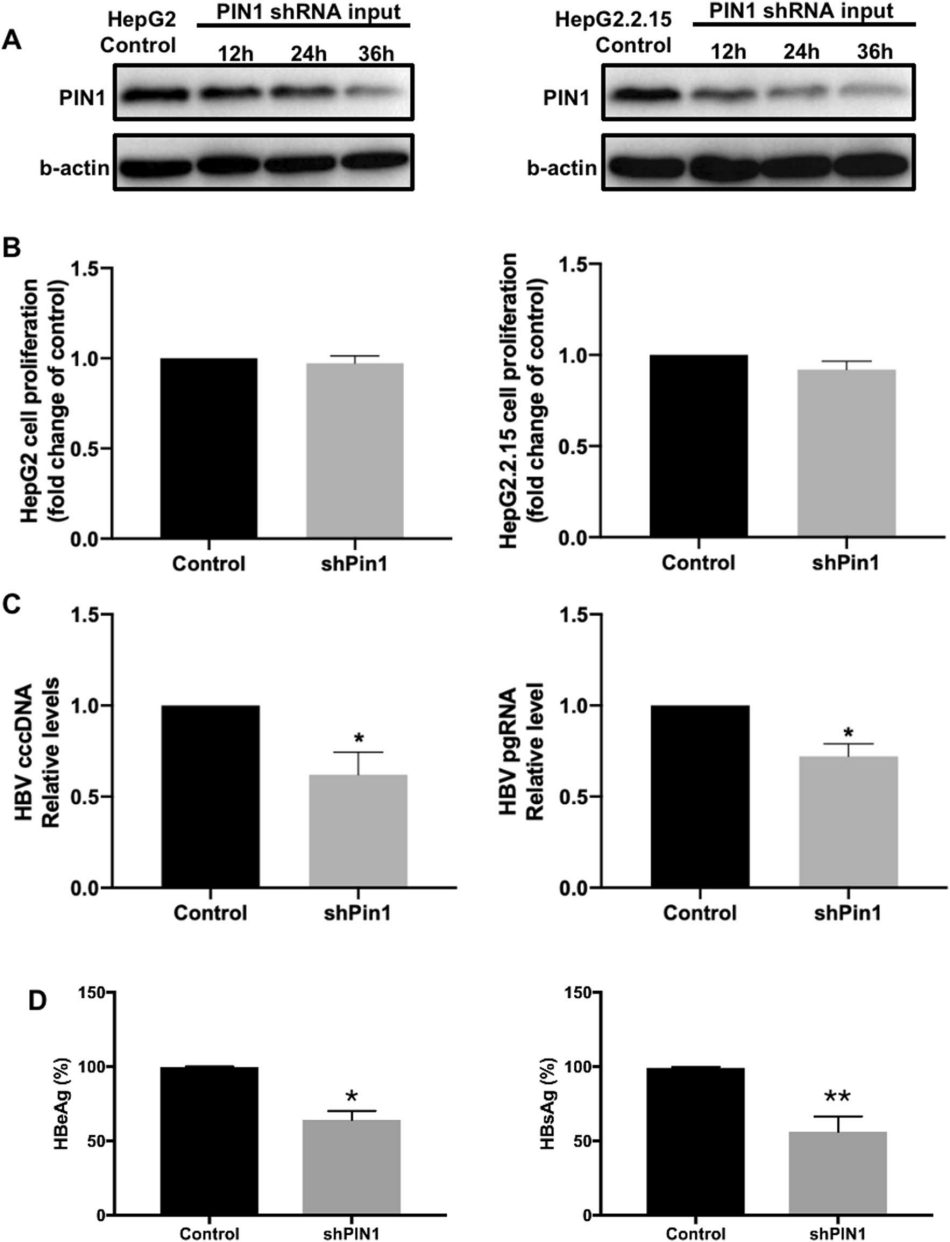
The role of Pin1 in HBV replication and HBx enhanced HBV replication. Levels of Pin1 expression detected by Western Blot after transfected with Pin1 shRNA for 12 h, 24 h, and 36 h in HepG2 and HepG2.2.15 cells (**A**). Cell proliferation of HepG2 and HepG2.2.15 cells were detected by CCK8 kit after transfected with Pin1 shRNA for 36 h and compared with un-treated control group (**B**). HepG2.2.15 cells were transfected with Pin1 shRNA for 36 h, levels of HBV cccDNA and pgRNA in cell-culture supernatant were detected by qPCR (**C**) and levels of HBeAg and HBsAg in cell culture supernatant were detected using ELISA test (**D**) and compared with control group. Data shown are the means ± SD from three parallel experiments. Statistical significance was examined by one-way analysis of variance pairwise comparison. *p* < 0.05 was considered statistically significant; *: *P* < 0.05; **: *P* < 0.01; ***: *P* < 0.001.

The expression of Pin1 was inhibited by shRNA-Pin1 in HepG2 and HepG2.2.15 cells. Western blot confirmed that the Pin1 expression value was significantly reduced with the transfection of shRNA-Pin1, while shRNA empty plasmids did not affect the Pin1 level comparing with control group (Fig. 4A). Considering that Pin1 was reported to enhance cell proliferation^22^, which could affect HBV propagation as HBV replication is dependent on cell cycle^23^, experiments investigating the effect of shRNA-Pin1 on cell proliferation were carried out. CCK8 results showed that there was no significant difference of cell proliferation between shRNA transfected cells and control group (Fig. 4B) in both HepG2 and HepG2.2.15 cells. The role of Pin1 in HBV transcription and expression was investigated by inhibiting Pin1 expression in HepG2.2.15 cells. shRNA-Pin1 transfection reduced the HBV cccDNA, HBV pgRNA, as well as HBeAg and HBsAg levels in HepG2.2.15 cell-culture supernatant (Fig. 4C,D), indicating that inhibition of Pin1 reduced the replication of HBV.

### Role of Pin1 in HBx augmenting HBV replication

**Figure 5:**
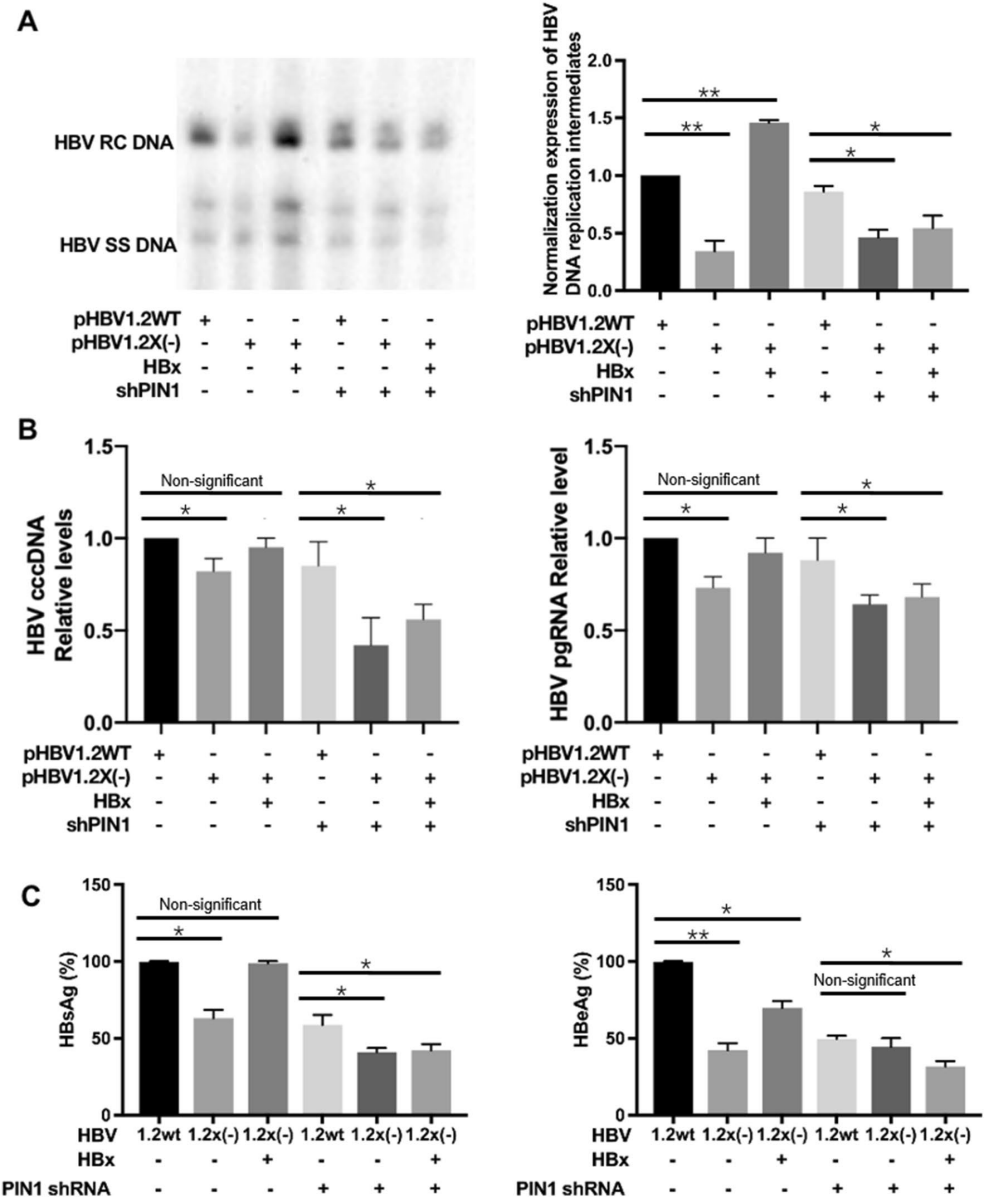
Role of Pin1 in HBx augmenting HBV replication. HepG2 cells were transfected with Pin1 shRNA for 36 h, and then transfected with wild-type HBV, or HBx-deficient HBV, with or without ectopic expression of HBx plasmid. Levels of HBV DNA replication intermediates detected by Southern Blot in HepG2 cells (**A**). Levels of HBV cccDNA and pgRNA in cell-culture supernatant were detected by qPCR (**B**). Levels of HBsAg and HBeAg (**C**) in cell-culture supernatant were detected by ELISA assay. Data shown are the means ± SD from three parallel experiments. Statistical significance was examined by one-way analysis of variance pairwise comparison. *p* < 0.05 was considered statistically significant; *: *P* < 0.05; **: *P* < 0.01; ***: *P* < 0.001.

In addition, the potential role of Pin1 expression in process of HBx augmenting HBV replication was further investigated in HepG2 cells. As shown by southern blotting in Fig. 5A, compared with wild-type HBV, the HBx-deficient HBV genome exhibited lower levels of HBV DNA replication intermediates which could be restored with the transfection of exogenous HBx expression plasmids, indicating that HBx participates in the transcription and replication of HBV with a positive regulative function. However, with the transfection of shRNA-Pin1, HBx-deficient virus exhibited lower levels of replication intermediates comparing with in wild-type HBV, which could not be restored even with the supplement of HBx expression. The levels of HBV cccDNA, HBV pgRNA, HBsAg and HBeAg in the cell-culture supernatant (Fig. 5B,C) exhibited the same trend as well. These results indicated that Pin1 might be associated with the function of HBx in HBV replication.

### Role of Pin1 in Wnt signaling involved in HBV replication

**Figure 6:**
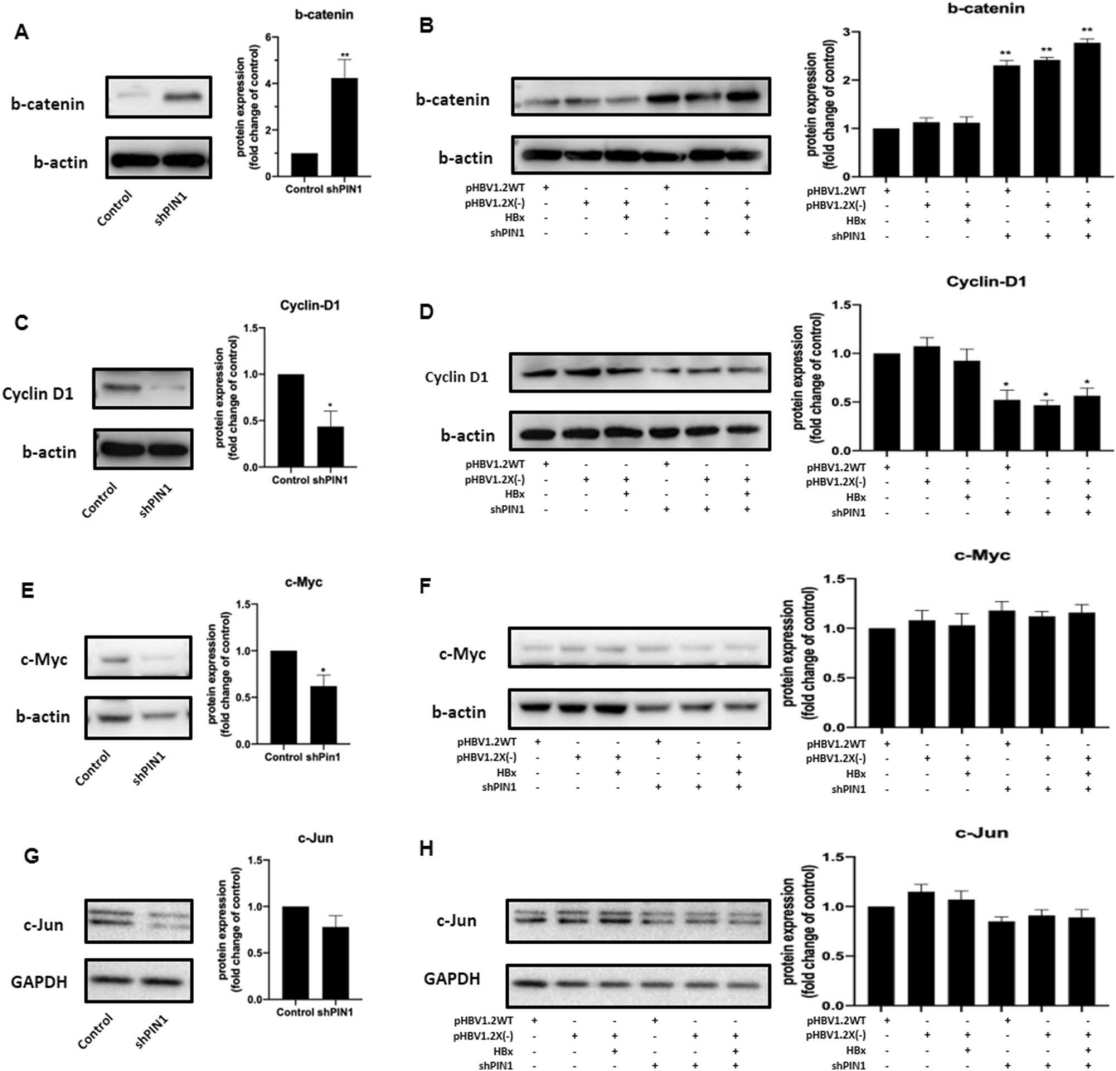
HepG2.2.15 cells were transfected with Pin1 shRNA for 36 h, and HepG2 cells were transfected with shPin1, wild-type HBV or HBx-deficient HBV, with or without exogenous expression of HBx plasmid. Levels of b-catenin, cyclin D1, c-Myc, and c-Jun expression were detected by Western Blot: b-catenin expression levels in HepG2.2.15 cells (**A**) and HepG2 cells (**B**); cyclin D1 expression levels in HepG2.2.15 cells (**C**) and HepG2 cells (**D**); c-Myc expression levels in HepG2.2.15 cells (**E**) and HepG2 cells (**F**); c-Jun expression levels in HepG2.2.15 cells (**G**) and HepG2 cells (**H**).

Considering that the HBx interactors identified in this study were significantly enriched in several biological process terms which have great correlation with Wnt signaling, combined with the important role that Pin1 participates in, the expression of several proteins that involved in Wnt signaling were further investigated in Pin1 shRNA transfected cells. Western blot results showed that the expression of b-catenin was induced in Pin1 shRNA transfected cells while the expression of cyclin D1 was reduced (Fig. 6A,C). However, HBx was not observed to participate in regulating the expression of b-catenin and cyclin D1 (Fig. 6B,D). Coincide with previously published studies which showed that Pin1 promotes cyclin D1 overexpression directly or through intranuclear accumulation of beta-catenin in cancer cells, our results indicated that Pin1 was involved in regulating cyclin D1 and beta-catenin expression in HBV replication models. However, HBx was not observed to participate in regulating the expression of b-catenin and cyclin D1.

Besides, expression of correlated proteins including c-Myc and c-Jun were also investigated in Pin1 shRNA transfected cells. Expression of both c-Myc and c-Jun were observed to reduce in HepG2.2.15 cells (Fig. 6E,G). However, no significant difference was observed HepG2 cells (Fig. 6F,H), indicating that the function of HBx was not dependent on these proteins. Further investigations into the detailed manner of HBx enhancing HBV replication are still needed in the future.

## Discussion

As an important determinant during the life cycle of HBV, HBx has been characterized to mediate the pathological effects of HBV according to interaction with various proteins in host cells^17^. Published studies have identified abundant proteins interact with HBx, such as jumonji C-domain-containing 5 (JMJD5)^24^, focal adhesion protein^25^, and Hsp40^26^, which participated in the HBV replication and development of HBV related hepatocellular carcinoma. However, this study is to our knowledge the first study to select the proteins that specifically interact with the transactivation domain of HBx, which can be regarded as the “key” host proteins that involved in the functions of HBx. According to GST pull-down and following MS analysis, our study identified 402 proteins interact with full-length HBx and 351 proteins interact with the N-terminal truncation of HBx which was not required in augmenting HBV replication. As a result, 189 proteins interact with the functional domain of HBx were further selected according to comparison, which are of great potential to affect the transcription and replication of HBx.

Previously published studies regarding the identification of HBx interacting proteins were mainly focused on yeast two-hybrid screen, which was criticized to have high possibility of both false positive and false negative identifications^27^. With the reduced false identification and improved sensitivity, tag-based pull-down assays conducted in vitro exhibited more advantages in researches of protein–protein interactions^28,29^. Even though expression microarray profiling and chromatin immunoprecipitation have also been applied to identify HBx target proteins in nuclear, which were mainly transcriptional regulators^19^, other proteins located in cytoplasm were likely missed. Consistent with previously published literatures that HBx located predominantly in cytoplasm^30^, our study also identified that more than half of the HBx interacted proteins located in cytoplasm (Supplementary Table 3). Considering that the interaction between proteins identified by modified target protein may introduce artificial deviations, the interactions need to be further validated. Among the 189 proteins identified in this study, there were 14 proteins reported to bind to HBx in other studies, such as DNA damage-binding protein 1 (DDB1)^31^, mitochondrial 60 kDa heat shock protein (HSPD1)^32^, peroxiredoxin-4 (PRDX4)^17^, and peptidyl-prolyl *cis*–*trans* isomerase (Pin1)^33^, which contributed to the reliability of the GST pull-down assay results.

HBx is easily to degrade with a short half-life^34,35^, which is also regarded as the barrier in the research of crystal structure of HBx and complex of its interacting proteins. The stability of the structure has been proved to associated with its transactivation ability^35,36^. Considering that Pin1 has been shown to bind to specific proteins and regulate its function according to stabilization of the targets, such as p53 and ß-catenin^37,38^, Pin1 may interact with HBx in a similar manner. Pang et al. has also identified the interaction between Pin1 and HBx by GST pull-down and Co-IP analysis, and provided evidence to the role of Pin1-HBx interaction in increasing the steady-state level of HBx and luciferase activity of HBx, as well as enhancing hepatocarcinogenesis in HBV-infected hepatocytes, which was relied on the interaction between the two proteins^22^. However, the effect of Pin1 in HBx augmenting HBV replication has not been defined yet. Based on their study, our study further confirmed the interaction between HBx and Pin1 in HepG2 cells, showed a significantly decreased HBsAg and HBeAg levels in Pin1 expression inhibited HepG2.2.15 cells, indicating that Pin1 has a potential function of regulating HBV replication. Besides, the HBV replication ability in HepG2 cells transfected with HBx-deficient HBV could be restored by ectopic HBx expression, but the inhibition of Pin1 expression could not be restored to wild-type of HBV anymore.

As a regulator participates in ß-catenin signaling and cyclin D1, the interaction between Pin1 and ß-catenin inhibits the degradation and transportation resulting the stabilization and accumulation of ß-catenin in nuclear and subsequent activation of transcriptional factors^37^. Besides, Pin1 can enhance the transcriptional activity of cyclin D1 by binding directly to cyclin D1 or activating the protein 1 (AP-1) site in promoter, to participate in the subcellular localization and stabilization of cyclin D1^39^. As described in previous studies, HBx participates in the transactivation of transcriptional factors or target genes such as c-myc, AP-1, and cyclin D1^16,40–42^, suggesting the similar pathway and common targets of Pin1 and HBx in molecular biological process, which is also of great potential to be further investigated in pathogenic of HBV and HCC. However, HBx was not observed to regulate the expression of cyclin D1 and ß-catenin in either control group or Pin1 inhibited cells in our study, as well as two other correlated proteins including c-Myc and c-Jun. Further studies investigating the mechanism of HBx regulating HBV replication are highly recommended in the future.

Recent study has also investigated the function of Pin1 on regulating HBV replication. Nishi et al.^43^ showed that shPin1 was associated with reduced expression of HBcAg and inhibited HBV replication in HepG2.2.15 cells, which is coincide with our results. They also demonstrated that Pin1 binds and stabilizes hepatitis B virus core protein (HBc) in a phosphorylation-dependent manner, and promotes the efficient viral propagation. However, according to their findings, Pin1 is very likely to participated in HBV replication through different manners. Other study proved that pin-1 enhances cell proliferation and also induces several proteins in the host cell, which could also be a possible pattern to regulating HBV propagation as it is critical that HBV replication is dependent on cell cycle. Although no influence of Pin1 on cell proliferation was observed in the current study, we showed that Pin1 might be associated with the function of HBx in HBV replication, which also provided new insights into further studies of HBV replication.

In addition to HBV, Pin1 has been proved to participate in the replication of other virus. Lim et al. showed that Pin1 interacts directly with NS5A and NS5B proteins of HCV and plays unique roles in HCV replication^44^. Shogo et al. has demonstrated that in the life cycle of HIV-1, the uncoating event of capsid core was promoted by virion incorporated extracellular signal-regulated kinase 2 (ERK2)^45^ and requires an interaction of the capsid protein with Pin1 at a specific phosphorylated Ser16-Pro17 motif, which also affect the replication efficiency of the virus^46^. Besides, Pin1 regulates Epstein-Barr virus (EBV) DNA replication through interacting with EBV DNA polymerase catalytic subunit^47^ and participates in epstein barr virus (EBV)-related nasopharyngeal carcinoma^48^. In addition, Pin1 has also been proved to modulate the replication and propagation of feline coronavirus (FCoV)^49^, and cyprinid herpesvirus 2 (CyHV-2)^50^. Given that most of the interactions between Pin1 and other proteins rely on the Thr/Ser-Pro motif, the binding site of Pin1 and HBx are of highly potential to be the Ser39-Pro40 or Ser144-Pro145 region. Even though Pang et al. demonstrated the binding at Ser39-Pro40 of HBx^22^, our study suggesting the interacting region was more likely to locate on the 51–154 amino acid of HBx. Thus, further researches regarding the binding sites of Pin1 and HBx are highly recommended in the future.

In conclusion, we identified 401 proteins bind to full-length HBx by GST pull-down assay combined with MS analysis, including 189 proteins interact specifically with the transactivation domain of HBx. The interaction between Pin1 and HBx was further confirmed, and Pin1 was suggested to be associated with the function of HBx in HBV replication.

## Materials and methods

### Plasmid constructions

The mammalian expression plasmids pNKF-HBx express full-length HBx, and pNKF-HBx-D5 express the truncated HBx containing N-terminal amino acids 1–50, which does not have the augmentation ability as full-length HBx in HBV replication. pGEX-6p-1-HBx and pGEX-6p-1-HBx-D5 plasmids were constructed using pNKF-HBx and pNKF-HBx-D5 as templates which were described previously and subcloned into pGEX-6P-1 (GE Healthcare, USA) with the forward primer 5’-TACGAATTCATGGCTGCTAGGGTGTGC-3’ for both the two plasmids, reverse primer 5’-GCGTCTAGATTAGGCAGAGGTGAAAAAGTTGC-3’ for pNKF-HBx and 5’-TATCTCGAGTTACCCGTGGTCGGCCGGAAC-3’ for pNKF-HBx-D5, respectively.

The plasmid payw1.2 (1.2 wt, subtype ayw) was used to investigate HBV replication, which has been described in previous study^12^ and contains 1.2 copies of the wild-type HBV genome. The HBx-minusmutant vector payw*7(1.2x( −)) was used to investigate the function of HBx in HBV replication, which was also described previously and contains 1.2 copies of HBx-minus HBV genome.

### Cell culture and transfection

The human hepatocellular carcinoma HepG2 cell line was cultured in Dulbecco's Modified Eagle Medium (DMEM) adding 10% fetal bovine serum (FBS), 1 mM glutamate, and 1% Penicillin–Streptomycin solution. The HepG2 derived cell line HepG2.2.15 with stable HBV replication function was cultured in DMEM supplemented with 1% Penicillin–Streptomycin, 10% FBS, and 100 ug/ml of G148. Cells were maintained at 37 °C with 5% CO_2_ and passaged every 3 days. Using lipofectamine reagent (Gibco, Invitrogen, USA), cells were transfected with plasmids according the protocol recommended by manufacturer. Both of the HepG2 and HepG2.2.15 cell lines were purchased from ATCC.

### Preparation of GST fusion proteins

pGEX-6p-1-HBx and pGEX-6p-1-HBx-D5 plasmids containing coding sequences for full-length HBx or HBx N-terminal truncation HBx-D5 were transformed into *E. coli.* strain BL21. Single colonies were grown on Luria–Bertani (LB) plate overnight at 37 °C and selected for enlarge cultivation in LB medium at 37 °C until the optical density (600 nM) reached 0.6 following induction with 0.1 mM isopropyl-β-D-thiogalactopyranoside (IPTG) overnight at 20 °C. Proteins were obtained by sonication of the cells and purified by agarose affinity chromatography, and then solubilized to glutathione-Sepharose beads (GE Healthcare, USA) according to the instructions provided by manufacturer.

### GST pull down assay

HBx and HBx-D5 proteins solubilized to glutathione-Sepharose beads were incubated with HepG2 cell lysate overnight at 4 °C with the GST protein as the negative control. 3 sets of parallel experiment were carried out at the same time. Beads were washed with respective incubation buffer to remove the unbound proteins. Proteins bound to the beads were collected in elution buffer by centrifugation. Protein samples obtained from the parallel experiments were mixed together and concentrated for SDS-PAGE separation followed by silver staining.

### In-gel digestion and MS/MS identification

Bands of each samples were cut out of the gel into 6 parts and decolored with 50 mM NaS_2_O_3_ and 15 mM K_3_Fe(CN)_6_ following dehydration with 100% acetonitrile (ACN). Samples were firstly incubated with 10 mM DTT in 25 mM ammonium bicarbonate (ABC) at 60 °C for 1 h and then with 55 mM indole-3-acetic acid (IAA) at room temperature for 45 min, following washing step with 50% ACN and 25 mM ABC. Proteins were digested by sequencing grade modified trypsin (Promega, USA) in 50 mM ABC at 37 °C for 12–16 h and extracted by double extraction with 75% ACN, 0.1% TFA. Samples were dried and redissolved in 0.1% fomic acid for mass spectrometry analysis.

### *Ni2* + *-NTA pulldown assay*

*E. Coli* BL21 strain were transfected with His_6_-tagged Pin1 and Flag-tagged HBx plasmids, respectively. Single colonies were grown on LB plate overnight at 37 °C and selected for enlarge cultivation in LB medium at 37 °C until the optical density (600 nM) reached 0.8 following induction with 0.4 mM IPTG overnight at 20 °C. Cells were sonicated with probe on ice and incubated with Ni^2+^-NTA beads to pull-down the His_6_-tagged Pin1 protein. The Ni^2+^-NTA beads were fist washed with low concentration of imidazole to remove the unbound proteins and then with high concentration of imidazole to competitively bond to beads instead of His_6_-tagged Pin1 protein. Cell lysates isolated from Ni^2+^-NTA beads eluent were collected for western blotting to detect HBx.

### Co-immunoprecipitation

HepG2 cells were transfected with Flag-HBx or Flag-control plasmids and harvested 24 h after transfection. Cells were incubated with lysis buffer (100 mM NaCl, 100 mM Tris–HCl, 10 mM EDTA, 1%NP-40, pH 8.0) on ice for 30 min and supernatant of protein samples were collected after centrifugation (14,000 rpm for 5 min at 4 °C). Protein A/G sepharose was dealt with anti-Pin1, anti-FLAG or nonimmune serum antibodies previously, and incubated with protein samples at 4 °C for 2 h with constant rotation for immunoprecipitation. Beads-protein mixture were washed 3 times, and protein complex were suspended in sodium dodecyl sulfate buffer for western blot detection.

### Immunofluorescence microscopy

HepG2 cells were transfected with or without exogenous Flag-HBx plasmids using lipofectamine reagent. Samples were harvested 24 h after the transfection and fixed in 4% paraformaldehyde fixative following washing step with PBS and blocking step with 5% BSA. Cells were first incubated with primary antibody in PBS overnight at 4 °C, and then incubated with fluorochrome-conjugated secondary antibody at room temperature for 1 h in dark. HBx and Pin1 staining were detected using confocal microscope with the filter sets of excitation/emission 488/30 nm for green signal and 554/70 nm for red signal, respectively.

### Cell proliferation

Cell Counting Kit-8 (CCK-8) assays (Beyotime, China) were performed to determine cell proliferation. Cells were seeded at a density of 1 × 10^5^ cells/well in 24-well culture plates overnight and transfected with or without exogenous HBx expression plasmids and Pin1 shRNA plasmids. The culture medium was replaced with 500 ul new medium containing 10% CCK-8 solution at 36 h post-transfection and incubated for 2 h. Absorbance of the CCK-8 solution was measured at 450 nm using a microplate reader (Bio-rad, USA).

### Western blotting

Concentration of protein samples were determined using Pierce BCA protein assay kit (Thermo Fisher Scienfitic, USA). Samples were separated by 10% SDS-PAGE and transferred to PVDF membrane, and proteins were detected by first and second antibodies (Cell Signal Technology, Germany) with Western blot.

### Enzyme-linked immunosorbent assay (ELISA)

HepG2 and HepG2.2.15 cells were cultured in 6-well plates and transfected with or without exogenous HBx expression plasmids or Pin1 shRNA plasmids 24 h after the cell seeding. The cell culture medium was collected 36 h after transfection, and ELISA test was performed for measurements of HBsAg and HBeAg level in the cell culture medium using ELISA kits (Kehua Bioengineering, China) according to the recommendation of the manufacturer. Briefly, cell-culture supernatant samples were collected 36 h after transfection, and incubated with coated plate for 1 h in 37 °C. HBsAg-HRP or HBeAg-HRP solutions were added to the plate and incubated for 30 min in the dark. Then the plate was washed using washing buffer and incubated with detection solution. Dual absorbance at 450 nm/630 nm was measured using a microplate reader (Bio-rad, USA).

### Detection of HBV cccDNA and pgRNA

pgRNA in cell-culture supernatant was detected by RT-PCR. Total RNA from the supernatants was extracted by using RNA extraction kit (Takara, China) and reverse transcribed using a PrimeScript™ RT reagent Kit to produce cDNA; RT-PCR was performed to detect pgRNA using Premix Ex Taq™ (Takara, China) using the primers 5’-CACCTCTGCCTAATCATC-3’ and 5’-GGAAAGAAGTCAGAAGGCAA-3’.

The cccDNA levels in cells were quantified by quantitative polymerase chain reaction (qPCR). Total DNA was extracted from the cells using a TIANamp Genomic DNA Kit (Tiangen, China) following DNase reagent (Takara, China) to enhance the efficiency of the specific extraction of cccDNA. qPCR was performed using primers 5’-GACTCCCCGTCTGTGCCTTCTCATC’, and 5’-AGACCAATTTATGCCTACAGCCTCC-3’ for cccDNA amplification using SYBR Premix Ex Taq (Takara, China).

Amplification was performed as follows: 95 °C for 30 s, 40 cycles of 95 °C for 5 s and 60 °C for 34 s, 95 °C for 15 s and 60 °C for 1 min.

### Southern blotting

HBV DNA replication intermediates were extracted and determined according to the protocol described in previous literatures^13^. Samples were resuspended in 30 μL of tris-ethylene diamine tetraacetic acid (TDTA) buffer and separated with 1% agarose gel. HBV replication intermediates samples were transferred to Hybond-N^+^ membrane (Amersham Biosciences, UK) and detected using digoxigenin-labeled full-length HBV DNA sequence as probe following further detection with the DIG Luminescent Detection Kit for Nucleic Acids (Roche, Germany).

### Bioinformatics and statistical analysis

The HBx PPI network was analyzed and built using the Cytoscape software^51^ (v3.8.2, Washington, USA, https://cytoscape.org/). Gene ontology analysis was conducted using the Database for Annotation, Visualization and Integrated Discovery (DAVID) v6.8 online tool (https://david.ncifcrf.gov/home.jsp).

SPSS software (V23, Chicago, USA) as well as GraphPad Prism (v8.1.3, San Diego, Ca) were used for conducting student’s t-test. *P* value < 0.05 was regarded as statistical significance.

### Statements of approval and accordance

No specific ethical approval was required for this study as no humans or in vivo animal experiments were involved in this study.

## Supplementary Information

Supplementary Information.
