# Supplementary material for: Screening of the HBx transactivation domain interacting proteins and the function of interactor Pin1 in HBV replication
Source: Sci Rep. 2021 Jul 8;11:14176. doi: 10.1038/s41598-021-93584-z (PMC8266847; doi:10.1038/s41598-021-93584-z)
Supplement: Supplementary file 1 — Supplementary Information. [file 41598_2021_93584_MOESM1_ESM.pdf]

Screening of the HBx transactivation domain interacting proteins and the function of interactor Pin1 in HBV replication

Authors and affiliations

Qiaoxia Zhou[1 §], Libo Yan[2,3 §], Baofu Xu[1,4], Xue'er Wang[1], Xuehong Sun[1], Ning Han[2,3], Hong Tang[2,3]*, Feijun Huang[1]*

[1] Department of Forensic Pathology, West China School of Preclinical and Forensic Medicine, Sichuan University, Chengdu 610041, People's Republic of China

[2] Center of Infectious Diseases, West China Hospital, Sichuan University, Chengdu 610041, People's Republic of China

[3] Division of Infectious Diseases, State Key Laboratory of Biotherapy, Sichuan University, Chengdu 610041, People's Republic of China

[4] Xinxiang Key Laboratory of Forensic Science Evidence, School of Forensic Medicine, Xinxiang Medical University, Xinxiang 453003, People's Republic of China

[§] These authors contributed equally to this work.

Running title: HBx transactivation domain interacting proteins

***Corresponding Author:**

Feijun Huang, M.D, Department of Forensic Pathology, West China School of Preclinical and Forensic Medicine, Sichuan University, No. 17 Third Renmin Road North, Chengdu 610041, People's Republic of China.

E-mail: fjhuang60123@hotmail.com

Tel: +86 13096326175

Hong Tang, M.D, Center of Infectious Diseases, West China Hospital of Sichuan University, No.37 Guo Xue Xiang, Chengdu 610041, People's Republic of China.

Tel: +862885422650;

Fax: +862885423052;

E-mail address: htang6198@hotmail.com

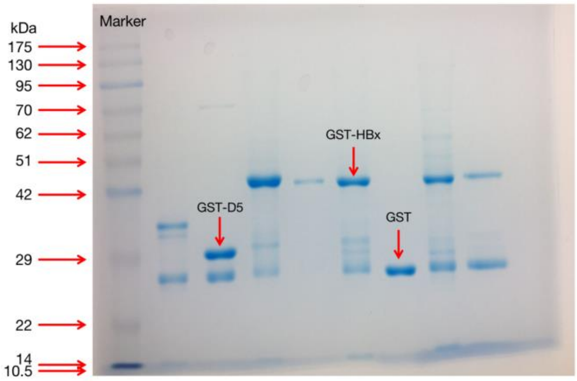

Supplementary Figure 1

SDS-PAGE blots of purified GST-fusion proteins (GST-HBx, GST-HBx-D5, GST). The purified proteins were loaded with 2 μg of equal weight, separated with 10% gel and stained with Coomassie brilliant blue.

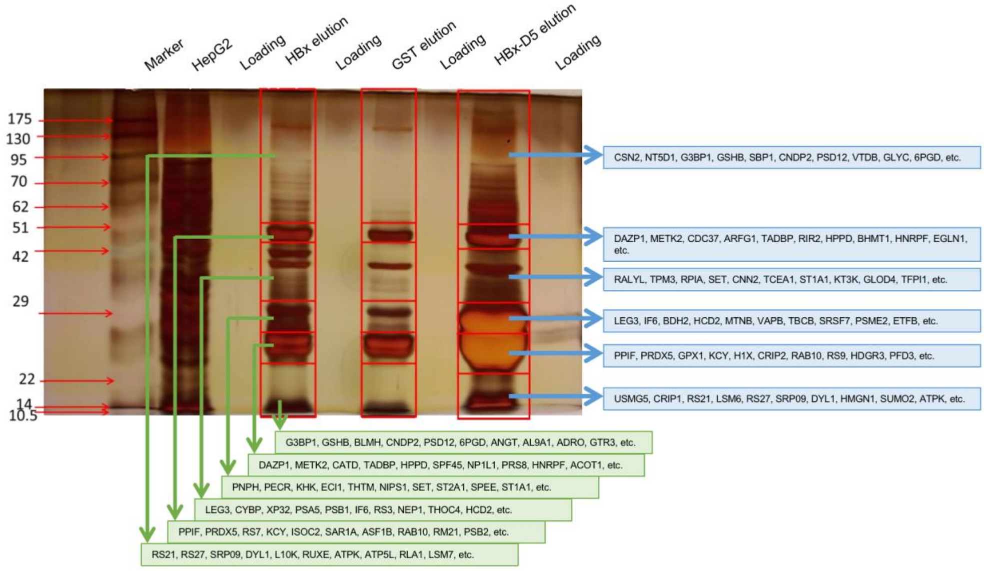

Supplementary Figure 2

Silver-stained SDS-PAGE of GST-fusion proteins (GST-HBx or GST-HBx-D5) incubated with HepG2 cell lysate. The arrows indicate the HBx-interacting proteins identified by mass spectrometry as listed in the bottom (HBx) or right (HBx-D5).

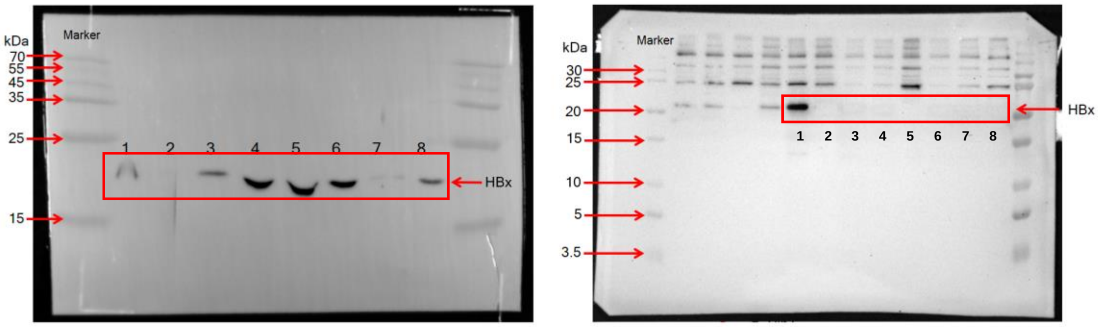

Supplementary Figure 3

Full-length blot for Figure 3A. Cell lysate of BL21 transfected with Flag-tagged HBx expression plasmids was incubated with Ni2+-NTA beads adding (left) or not adding (right) purified His6-tagged Pin1. HBx was detected in both the whole cell lysate isolated from Ni2+-NTA beads and imidazole eluent. Lane 1 to lane 8 were: (1) cell lysate after incubating with purified Pin1 and Ni2+-NTA; (2) low concentration of imidazole elution for removing non-specific binding bacteria proteins; (3-8) high concentration of imidazole elution for removing His6-tagged Pin1 and its binding proteins including HBx.

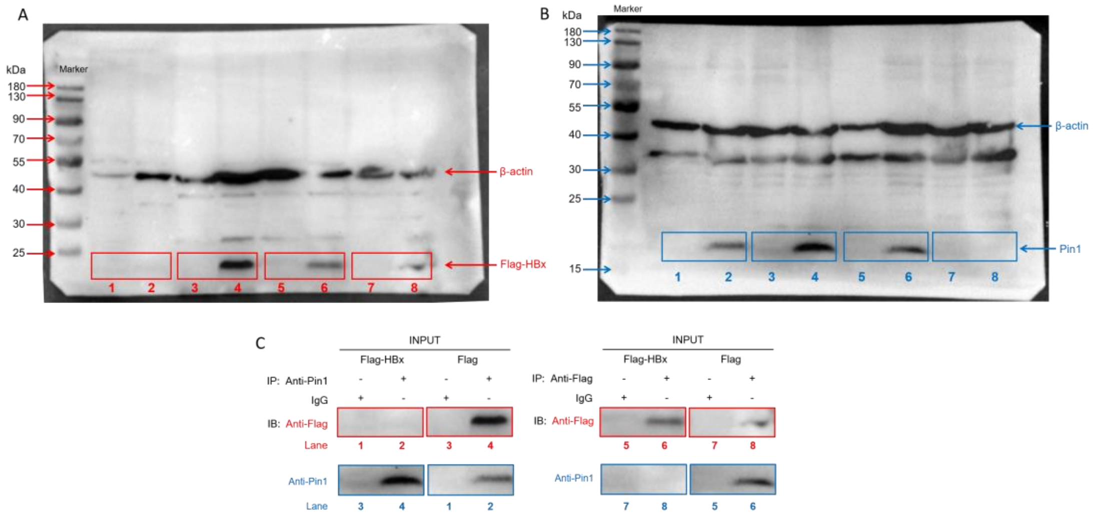

Supplementary Figure 4

Full-length blot for Figure 3B. HepG2 cells were transfected with Flag-HBx or Flag-control plasmids and harvested 24 hours after transfection. Immunoprecipitation was carried out using protein A/G sepharose dealt with anti-Pin1, anti-FLAG or nonimmune serum antibodies previously, and incubated with protein samples for immunoprecipitation. Flag-HBx or Pin1 pulled down as protein complex was detected using western blot. Co-immunoprecipitation assay results showed that with the transfection of Flag-tagged HBx, HBx was detected in eluent after incubated with anti-Pin1 beads (Fig. S4A and blots with red border in Fig. S4C), while endogenous Pin1 protein pulled down by HBx was detected in eluent after incubated with anti-Flag beads (Fig. S4B and blots with blue border in Fig. S4C). Fig. S4C summarized the cropped blots and marked with lane numbers.

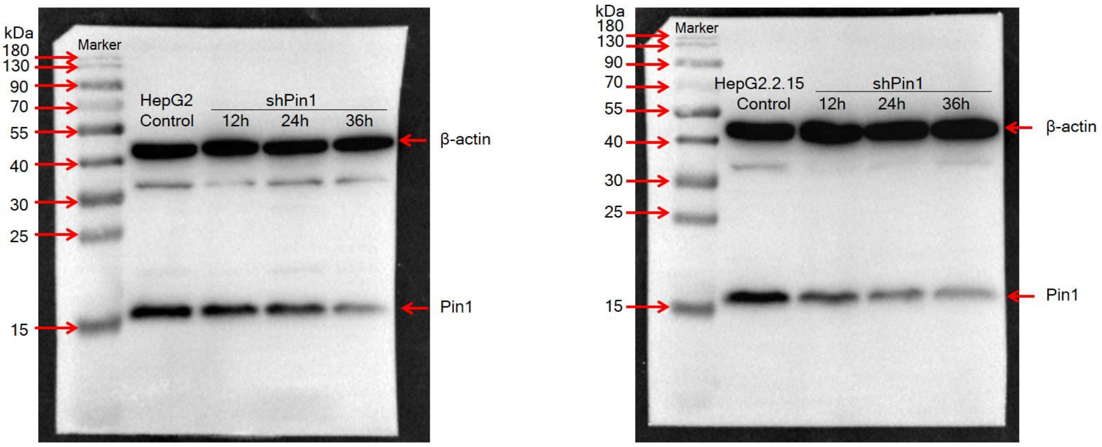

Supplementary Figure 5
Full-length blot for Figure 4A. Levels of Pin1 expression detected by Western Blot after transfected with Pin1 shRNA for 12h, 24h, and 36h in HepG2 (left) and HepG2.2.15 cells (right).

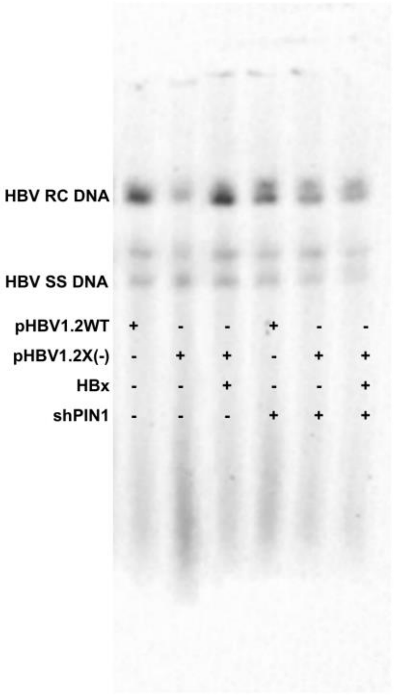

Supplementary Figure 6
Full-length blot for Figure 5A. Levels of HBV DNA replication intermediates detected by Southern Blot in HepG2 cells which were transfected with wild-type HBV, or HBx-deficient HBV, with or without exogenous expression of HBx plasmid.

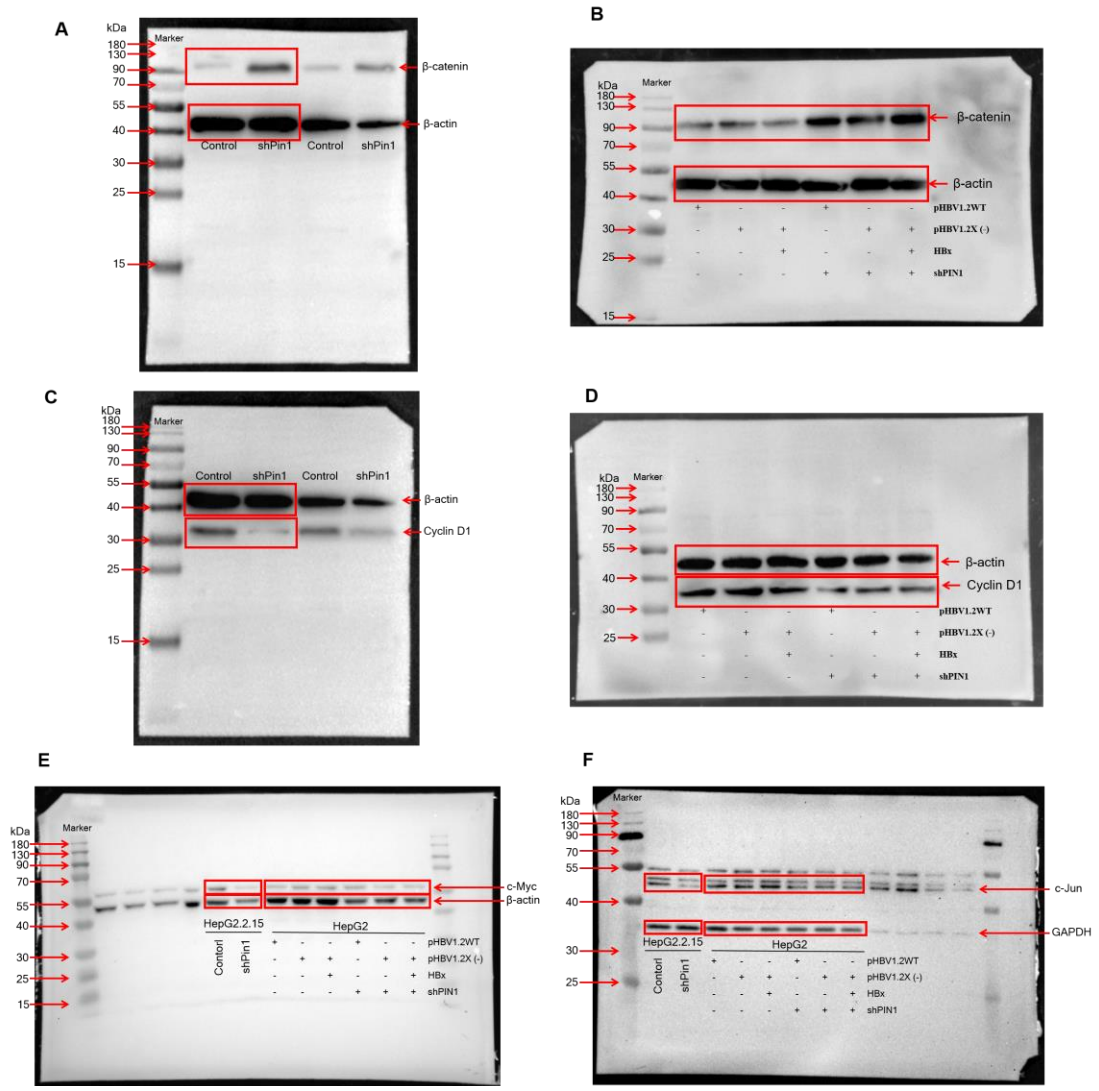

Supplementary Figure 7

Full-length blot for Figure 6. HepG2.2.15 cells were transfected with Pin1 shRNA for 36h, and HepG2 cells were transfected with shPin1, wild-type HBV or HBx-deficient HBV, with or without exogenous expression of HBx plasmid. Levels of b-catenin, cyclin D1, c-Myc, and c-Jun expression were detected by Western Blot: A. b-catenin expression level in HepG2.2.15 cells, full-length blot of Fig. 6A; B. b-catenin expression level in HepG2 cells, full-length blot of Fig. 6B; C. cyclin D1 expression level in HepG2.2.15 cells, full-length blot of Fig. 6C; D. cyclin D1 expression level in HepG2 cells, full-length blot of Fig. 6D; E. c-Myc expression level in HepG2.2.15 cells or HepG2 cells, full-length blot of Fig. 6E and 6F; F. c-Jun expression level in HepG2.2.15 cells or HepG2 cells, full-length blot of Fig. 6G and 6H.

Supplementary Table 1.

Supplementary Table 1. Biological process by GO analysis of the proteins specifically interacting with the transactivation domain of HBx

Category	Count	%	PValue	Top 10 Genes	Fold Enrichme
regulation of cellular amino acid metabolic process	15	7.89473684	1.79E-16	P43686, P25789, P25786, P25787, Q99460, P62195, P20618, P28072, P28070, O14818, I	26.8414322
regulation of mRNA stability	18	9.47368421	9.85E-16	P43686, P25789, P25786, Q5RKV6, P25787, P39687, Q99460, P62195, P20618, P28072,	15.9485015
antigen processing and presentation of exogenous peptide antigen via MHC class I,	15	7.89473684	4.69E-15	P43686, P25789, P25786, P25787, Q99460, P62195, P20618, P28072, P28070, O14818, I	21.7287785
NIK/NF-kappaB signaling	15	7.89473684	9.53E-15	P43686, P25789, P25786, P25787, Q99460, P62195, P20618, P28072, P28070, O14818, I	20.7411067
positive regulation of canonical Wnt signaling pathway	18	9.47368421	1.46E-14	P43686, P25789, P25786, P25787, Q66K66, Q99460, P14923, P62195, P20618, P28072, I	13.6891304
negative regulation of ubiquitin-protein ligase activity involved in mitotic cell cycle	15	7.89473684	2.83E-14	P43686, P25789, P25786, P25787, Q99460, P62195, P20618, P28072, P28070, O14818, I	19.2804654
Wnt signaling pathway, planar cell polarity pathway	16	8.42105263	6.42E-14	P43686, P25789, P25786, P25787, Q99460, P62195, P20618, P28072, P28070, O14818, (	15.8714556
positive regulation of ubiquitin-protein ligase activity involved in regulation of mitoti	15	7.89473684	7.70E-14	P43686, P25789, P25786, P25787, Q99460, P62195, P20618, P28072, P28070, O14818, I	18.0120137
anaphase-promoting complex-dependent catabolic process	15	7.89473684	1.36E-13	P43686, P25789, P25786, P25787, Q99460, P62195, P20618, P28072, P28070, O14818, I	17.3280132
stimulatory C-type lectin receptor signaling pathway	15	7.89473684	7.81E-12	P43686, P25789, P25786, P25787, Q99460, P62195, P20618, P28072, P28070, O14818, I	13.0372671
tumor necrosis factor-mediated signaling pathway	15	7.89473684	3.93E-11	P43686, P25789, P25786, P25787, Q99460, P62195, P20618, P28072, P28070, O14818, I	11.600958
protein polyubiquitination	17	8.94736842	1.75E-10	P43686, P25789, P25786, P25787, Q13867, Q99460, P62195, P20618, P28072, P28070, (	8.43171078
negative regulation of canonical Wnt signaling pathway	16	8.42105263	3.00E-10	P43686, P25789, P25786, P25787, Q99460, P62195, P20618, P28072, P02649, P28070, (	8.95812217
T cell receptor signaling pathway	15	7.89473684	8.34E-10	P43686, P25789, P25786, P25787, Q99460, P62195, P20618, P28072, P28070, O14818, I	9.24941246
Fc-epsilon receptor signaling pathway	16	8.42105263	1.03E-09	P43686, P25789, P25786, P25787, Q99460, P62195, P20618, P28072, P62993, P28070, (	8.20322423
proteolysis involved in cellular protein catabolic process	10	5.26315789	2.07E-09	P20618, P28072, P25789, P25786, P28070, P25787, O14818, P60900, P49721, P49720	19.0126812
MAPK cascade	17	8.94736842	2.94E-08	P43686, P25789, P25786, P25787, Q99460, P62195, Q9UHA4, P20618, P28072, P62993,	5.9215068
proteasome-mediated ubiquitin-dependent protein catabolic process	15	7.89473684	4.93E-08	P43686, P25789, P25786, P25787, Q99460, P62195, P20618, P28072, P28070, O14818, I	6.74341401
nuclear-transcribed mRNA catabolic process, nonsense-mediated decay	11	5.78947368	7.04E-07	P62280, P83881, P05386, P67775, P62906, P62266, P62081, P61353, P15170, P23396, P	8.4358787
viral process	16	8.42105263	1.01E-06	Q7L5N1, P25789, Q9Y490, P14324, P20618, O95373, Q15046, Q7KZF4, P28072, P62993	4.88352479
translational initiation	11	5.78947368	2.56E-06	P62280, P83881, P05386, P62906, P62266, P62081, Q14152, P61353, P23396, P46782, F	7.32751507
viral transcription	10	5.26315789	3.73E-06	P62280, P83881, P05386, P62906, P62266, P62081, P61353, Q9NRG9, P23396, P46782	8.14829193
rRNA processing	13	6.84210526	4.07E-06	P62280, P83881, Q5RKV6, P05386, Q14137, Q15024, P62906, P62266, P62081, P61353,	5.5438846
SRP-dependent cotranslational protein targeting to membrane	9	4.73684211	8.55E-06	P62280, P83881, P05386, P62906, P62266, P62081, P61353, P23396, P46782	8.73774283
translation	12	6.31578947	1.05E-04	P62280, P83881, P05386, P26639, P62906, P62266, P62081, Q7Z2W9, Q9Y3D3, P61353,	4.32857879
nucleosome assembly	8	4.21052632	3.27E-04	Q15020, Q9BTT0, Q9Y294, P55209, P39687, Q9NVP2, Q5SSJ5, Q92688	6.13518451
cell-cell adhesion	11	5.78947368	7.95E-04	Q8WWM7, Q7KZF4, P28072, Q9UHX1, Q96AG4, Q15691, P31947, O00571, P25685, O1	3.70431574
osteoblast differentiation	7	3.68421053	9.68E-04	P62280, P84090, Q7KZF4, P35232, Q9Y294, Q86V81, Q00610	6.14255853
cell redox homeostasis	6	3.15789474	0.00152553	P02788, O95881, Q8NBF2, P09622, Q6DKJ4, Q13162	7.11123659
mRNA splicing, via spliceosome	9	4.73684211	0.00304773	Q07955, Q15020, P52434, Q86V81, O43390, Q9UK45, Q9Y3B4, P31943, P62304	3.69976498
negative regulation of protein catabolic process	4	2.10526316	0.00500599	P35232, Q13526, P21333, Q99497	11.4076087
platelet degranulation	6	3.15789474	0.00539019	P02763, Q9Y490, P01009, P21333, P04217, P01011	5.31616716
histone exchange	3	1.57894737	0.00608992	Q9BTT0, P39687, Q92688	24.8893281
mitotic spindle assembly	4	2.10526316	0.00698072	Q15018, P21333, Q00610, P62491	10.1400966
acute-phase response	4	2.10526316	0.00872499	P02763, P01009, P00734, P01011	9.36008919
proteolysis	13	6.84210526	0.00880313	O43464, P43686, P01861, P00751, P02788, Q13867, P55786, P12273, P07339, P09622,	2.37278261
ceramide metabolic process	3	1.57894737	0.01442718	Q9Y5P4, O43464, P67775	16.1048593
nicotinamide riboside catabolic process	2	1.05263158	0.02167797	P00491, Q13126	91.2608696
oxidation-reduction process	13	6.84210526	0.0289985	O00469, P12268, Q8N4T8, O14618, Q13162, P11586, Q8IZ83, Q9BY49, O95881, P1512	2.00403937
nucleocytoplasmic transport	3	1.57894737	0.03006841	Q9BTT0, P39687, Q9NRG9	10.9513043
positive regulation of B cell activation	3	1.57894737	0.03234473	P01861, P01871, P01877	10.5301003
phagocytosis, recognition	3	1.57894737	0.03709217	P01861, P01871, P01877	9.77795031
exonucleolytic nuclear-transcribed mRNA catabolic process involved in deadenylatic	3	1.57894737	0.03955995	Q5RKV6, Q15024, Q9UK45	9.44077961
cellular oxidant detoxification	4	2.10526316	0.04096168	P02649, Q6DKJ4, Q13162, Q99497	5.21490683
positive regulation of protein insertion into mitochondrial membrane involved in apc	3	1.57894737	0.04208828	P27348, P31947, Q04917	9.12608696
positive regulation of reactive oxygen species metabolic process	3	1.57894737	0.04208828	P62993, P01019, P00734	9.12608696
intracellular protein transport	7	3.68421053	0.04465578	Q9NR31, O95373, Q04917, Q00610, Q10567, P30040, Q4G0F5	2.7068902
membrane organization	3	1.57894737	0.04732018	P27348, P31947, Q04917	8.55570652
protein folding	6	3.15789474	0.04735204	O75347, Q14697, O95816, P67870, P25685, P30040	3.04202899
ubiquitin-dependent protein catabolic process	6	3.15789474	0.04919666	P25789, P25786, P25787, O14818, P60900, O00233	3.0086001
regulation of immune system process	2	1.05263158	0.05332164	P02763, P12273	36.5043478
transsulfuration	2	1.05263158	0.05332164	P25325, P11586	36.5043478
phagocytosis, engulfment	3	1.57894737	0.05558303	P01861, P01871, P01877	7.82236025
positive regulation by host of viral process	2	1.05263158	0.06364166	P11498, P02649	30.4202899
positive regulation of gene expression	7	3.68421053	0.06744683	P35232, P12273, O00571, P30040, P23396, Q99497, P34913	2.43826751
retina homeostasis	3	1.57894737	0.070363	P02788, P01877, P12273	6.84456522
negative regulation of neuron death	3	1.57894737	0.070363	O43464, P02649, Q99497	6.84456522
protein catabolic process	3	1.57894737	0.07345766	P43686, P07339, P62195	6.6776246
negative regulation of protein ubiquitination	3	1.57894737	0.07345766	P23396, Q6DKJ4, Q99497	6.6776246
modulation by virus of host process	2	1.05263158	0.07384979	O15131, O00505	26.0745342
negative regulation of inflammatory response to antigenic stimulus	2	1.05263158	0.07384979	P25786, P28070	26.0745342
antibacterial humoral response	3	1.57894737	0.08299504	P02788, P01871, P01877	6.22233202
doxorubicin metabolic process	2	1.05263158	0.08394722	Q8N4T8, P15121	22.8152174
negative regulation of platelet activation	2	1.05263158	0.08394722	P02649, P00734	22.8152174
U4 snRNA 3'-end processing	2	1.05263158	0.08394722	Q5RKV6, Q15024	22.8152174
positive regulation of actin filament depolymerization	2	1.05263158	0.08394722	Q9Y281, Q96GD0	22.8152174
daunorubicin metabolic process	2	1.05263158	0.08394722	Q8N4T8, P15121	22.8152174
nuclear mRNA surveillance	2	1.05263158	0.09393517	Q5RKV6, Q15024	20.2801932
catecholamine metabolic process	2	1.05263158	0.09393517	P0DMM9, P21964	20.2801932
nuclear-transcribed mRNA catabolic process, exonucleolytic, 3'-5'	2	1.05263158	0.09393517	Q5RKV6, Q15024	20.2801932
positive regulation of cholesterol esterification	2	1.05263158	0.09393517	P02649, P01019	20.2801932
positive regulation of RNA polymerase II transcriptional preinitiation complex asseml	2	1.05263158	0.09393517	P43686, P62195	20.2801932
gene expression	3	1.57894737	0.09625594	P52434, O43390, P31943	5.70380435
substantia nigra development	3	1.57894737	0.09966034	P24539, P27348, Q04917	5.58740018
nucleobase-containing compound metabolic process	3	1.57894737	0.09966034	P84090, P00491, Q13126	5.58740018

Supplementary Table 2.

Supplementary Table 2. Molecular function by GO analysis of the proteins specifically interacting with the transactivation domain of HBx

Category	Count	%	PValue	Genes	Fold Enrichme
threonine-type endopeptidase activity	11	5.78947368	4.93E-15	P20618, P28072, P25789, P25786, P28070, P25787, O14818, P60900, P49721, P49720, P	48.5847724
poly(A) RNA binding	43	22.6315789	6.97E-13	P39687, O00571, P61353, P31943, P14324, P62280, Q7KZF4, P62993, Q9UHX1, Q14137	3.53265556
protein binding	131	68.9473684	3.75E-08	O15131, Q969U7, P19623, Q13867, Q5H9R7, P12268, O00571, P21964, Q14137, P1227	1.3831087
RNA binding	22	11.5789474	4.36E-07	Q9H361, P25786, P26639, O00571, P12268, Q96I25, Q14152, P62081, Q92667, P31943,	3.73045784
cadherin binding involved in cell-cell adhesion	14	7.36842105	1.53E-05	Q9Y490, P31947, O00571, P21333, P25685, P14923, O14618, Q8WWM7, Q7KZF4, P280	4.47771883
structural constituent of ribosome	11	5.78947368	1.44E-04	P62280, P83881, P05386, P62906, P62266, P62081, Q7Z2W9, Q9Y3D3, P61353, P23396,	4.59585685
histone binding	7	3.68421053	0.00202482	O95373, Q15020, Q9BTT0, Q9Y294, P39687, Q9NVP2, Q92688	5.32187894
protein transporter activity	5	2.63157895	0.00746388	O15131, O95373, Q10567, O00505, Q4G0F5	6.441163
phosphoprotein phosphatase activity	4	2.10526316	0.01236817	O00743, P62140, P67775, Q96GD0	8.24468864
nucleotide binding	10	5.26315789	0.01281505	Q9H361, Q07955, Q15020, Q9UHX1, Q86V81, O43390, Q96I25, P12268, Q9Y3B4, P319	2.66530883
catalytic activity	7	3.68421053	0.01594549	P00491, P46020, P42126, Q96AB3, P12268, P34913, Q13126	3.45355974
protein homodimerization activity	15	7.89473684	0.02568084	P19623, Q8WUM4, P26639, P21333, P14923, P68402, P30040, Q13162, P34913, P0264	1.90587837
immunoglobulin receptor binding	3	1.57894737	0.0313937	P01861, P01871, P01877	10.7022401
superoxide dismutase copper chaperone activity	2	1.05263158	0.03182455	O14618, Q99497	61.8351648
proteinase activated receptor binding	2	1.05263158	0.03182455	P35232, Q8WUM4	61.8351648
ubiquitin-like protein conjugating enzyme binding	2	1.05263158	0.04220728	P23396, Q99497	46.3763736
phosphoserine phosphatase activity	2	1.05263158	0.04220728	Q96GD0, P78330	46.3763736
mRNA binding	5	2.63157895	0.0456348	Q07955, Q14152, P23396, Q99497, P46782	3.71010989
hydrolase activity	6	3.15789474	0.05505521	P43686, P62140, P68402, Q9UKK9, P11586, P34913	2.91369887
chaperone binding	4	2.10526316	0.05636982	O75347, O95816, P25685, P30040	4.58038258
peroxiredoxin activity	2	1.05263158	0.06264171	Q13162, Q99497	30.9175824
proteasome-activating ATPase activity	2	1.05263158	0.06264171	P43686, P62195	30.9175824
oxidoreductase activity	6	3.15789474	0.06468813	P13804, Q8N4T8, P15121, P12268, Q13162, P37268	2.78258242
phosphatase activity	3	1.57894737	0.06840055	P62140, Q96GD0, P34913	6.95645604
rRNA binding	3	1.57894737	0.06840055	P62280, P46782, Q92979	6.95645604
magnesium ion binding	6	3.15789474	0.06884977	P36871, Q96GD0, P21964, Q9UKK9, P78330, P34913	2.72802198
serine-type endopeptidase inhibitor activity	4	2.10526316	0.08628548	P01019, P01009, P19827, P01011	3.82485556
transmembrane transporter activity	3	1.57894737	0.09697556	P24539, P11169, P48047	5.67873963

Supplementary Table 3.

Supplementary Table 3. Cellular component by GO analysis of the proteins specifically interacting with the transactivation domain of HBx

Category	Count	%	PValue	Genes	Fold Enrichme
extracellular exosome	110	57.8947368	1.56E-41	Q13867, P42126, O00571, P12268, P21964, P04217, P34913, Q7Z794, P38606, P15153,	3.79330442
cytosol	99	52.1052632	2.19E-26	O15131, Q969U7, Q13867, P19623, Q5H9R7, P12268, P21964, P34913, P14324, P3860	2.89492635
proteasome complex	15	7.89473684	9.79E-16	P43686, P25789, Q969U7, P25786, P25787, Q99460, P62195, P20618, P28072, P28070,	24.2340426
proteasome core complex	11	5.78947368	3.22E-15	P20618, P28072, P25789, P25786, P28070, P25787, O14818, P60900, P49721, P49720, P	50.7760892
cytoplasm	94	49.4736842	8.32E-10	O15131, Q13867, Q5H9R7, P31947, P12268, O00571, O14618, P31943, P31944, P3491	1.74492532
proteasome core complex, alpha-subunit complex	6	3.15789474	5.89E-09	P25789, P25786, P25787, O14818, P60900, P28066	72.7021277
nucleoplasm	60	31.5789474	1.56E-08	O15131, Q5H9R7, Q99460, P31943, P14324, P62280, P62993, Q14137, Q13526, O4339	2.0891416
blood microparticle	13	6.84210526	5.57E-08	P02763, P01861, P01871, P01877, P19827, P04217, P01011, P62195, P00751, P02649, P	8.29059351
mitochondrion	36	18.9473684	2.16E-07	Q9BPW8, P42126, P31947, P21964, P09622, Q13162, P11586, Q9BY49, O43464, P14324	2.62186486
extracellular matrix	14	7.36842105	1.21E-05	P62081, P21333, P14923, P61353, P31944, P68371, P62280, P02649, Q14697, P48047, P	4.58481886
focal adhesion	16	8.42105263	1.29E-05	Q9Y490, P62140, P05386, Q8WUM4, P62081, P21333, P14923, P61353, Q9UHA4, P622	3.9666975
nucleus	83	43.6842105	2.69E-05	O15131, Q969U7, Q13867, P31947, P12268, O00571, Q99460, O14618, P61353, P3194	1.48581757
cell-cell adherens junction	14	7.36842105	3.03E-05	Q9Y490, P31947, O00571, P21333, P25685, P14923, O14618, Q8WWM7, Q7KZF4, P280	4.20156775
ribosome	10	5.26315789	5.52E-05	P62280, P83881, P05386, P62906, P62266, P62081, Q7Z2W9, Q9Y3D3, P61353, P23396	5.83952833
melanosome	8	4.21052632	8.17E-05	Q7KZF4, Q14697, P55209, Q8WUM4, A6NMY6, P07339, Q00610, P30040	7.67811249
membrane	42	22.1052632	9.80E-05	P43686, P12268, P21964, Q99460, P61353, P31943, P11586, P62195, O43464, P62280,	1.85059961
cytosolic small ribosomal subunit	6	3.15789474	1.29E-04	P62280, P62266, O00571, P62081, P23396, P46782	12.1170213
proteasome regulatory particle, base subcomplex	4	2.10526316	2.18E-04	P43686, Q99460, O00233, P62195	32.3120567
catalytic step 2 spliceosome	7	3.68421053	3.70E-04	Q07955, Q86V81, O43390, Q9UK45, Q9Y3B4, P31943, P62304	7.37557817
intracellular ribonucleoprotein complex	8	4.21052632	5.13E-04	P42704, Q9UHX1, Q14137, O43390, P62081, P61353, P23396, P46782	5.70212766
mitochondrial matrix	11	5.78947368	0.00208315	P11498, P24539, Q15046, P43897, P30084, P42126, P13804, Q8N4T8, P09622, P23396,	3.26084976
nucleolus	19	10	0.00318698	O75347, Q5RKV6, P62140, Q14152, P62081, P21333, P25685, P68402, Q92688, P62280	2.14910996
myelin sheath	7	3.68421053	0.00485447	P24539, P35232, P38606, Q8WUM4, Q00610, P09622, P68371	4.46416573
proteasome regulatory particle	3	1.57894737	0.00541995	Q99460, O00233, P62195	26.4371373
proteasome accessory complex	3	1.57894737	0.01287187	P43686, Q99460, P62195	17.106383
extracellular vesicle	4	2.10526316	0.01463725	P02649, Q8WUM4, Q00610, P68371	7.75489362
platelet alpha granule lumen	4	2.10526316	0.01887872	P02763, P01009, P04217, P01011	7.04990329
actin cytoskeleton	7	3.68421053	0.02519901	P36871, Q9Y281, P26639, P21333, Q96GD0, P14923, P49720	3.11262932
U12-type spliceosomal complex	3	1.57894737	0.02896392	Q9UK45, Q9Y3B4, P62304	11.1849427
Myb complex	2	1.05263158	0.03047044	P21333, Q00610	64.6241135
small ribosomal subunit	3	1.57894737	0.03107351	P62266, P23396, P46782	10.7706856
cytosolic large ribosomal subunit	4	2.10526316	0.03276248	P83881, P05386, P62906, P61353	5.70212766
nuclear pore	4	2.10526316	0.03785899	O15131, O95373, Q9NRG9, O00505	5.38534279
cytoplasmic vesicle membrane	5	2.63157895	0.04093959	P27348, P31947, Q04917, Q10567, P62491	3.84667342
nuclear speck	6	3.15789474	0.05656607	Q07955, Q8WWM7, Q15020, Q86V81, Q13526, O00571	2.89361702
polysome	3	1.57894737	0.05786349	P25786, P60900, P23396	7.65285554
spliceosomal complex	4	2.10526316	0.07247007	Q9BZZ5, O43390, Q96I25, P62304	4.12494341
nuclear proteasome complex	2	1.05263158	0.07921578	P43686, P62195	24.2340426
cell	4	2.10526316	0.08370648	P02649, Q8NBF2, Q6DKJ4, Q13162	3.87744681
cytoskeleton	8	4.21052632	0.08863626	O43464, Q7Z794, P00491, P42704, P52565, Q96GD0, P14923, P68371	2.09026782
neuron projection	6	3.15789474	0.09738294	P25325, P16949, Q13526, Q9HB71, Q99497, P78330	2.45408026
cytosolic proteasome complex	2	1.05263158	0.09802425	P43686, P62195	19.387234
extracellular space	20	10.5263158	0.09816487	P02763, P01861, P02788, P01871, Q9Y281, P01877, P52565, P31947, P04217, Q13162,	1.43928983
